# DNA methylation and miR‐92a‐3p‐mediated repression of HIP1R promotes pancreatic cancer progression by activating the PI3K/AKT pathway

**DOI:** 10.1111/jcmm.17612

**Published:** 2023-02-21

**Authors:** Sixian Zhu, Huiting Xu, Runzhi Chen, Qian Shen, Dongmei Yang, Hui Peng, Jin Tong, Qiang Fu

**Affiliations:** ^1^ Department of Oncology, Tongji Hospital, Tongji Medical College Huazhong University of Science and Technology Wuhan China; ^2^ Department of Abdominal Oncology, Hubei Cancer Hospital Wuhan China; ^3^ Department of PICC, Tongji Hospital, Tongji Medical College Huazhong University of Science and Technology Wuhan China

**Keywords:** DNA methylation, HIP1R, miR‐92a‐3p, pancreatic cancer (PAAD), PI3K/AKT

## Abstract

Pancreatic cancer (PAAD) is a highly malignant tumour characterized of high mortality and poor prognosis. Huntingtin‐interacting protein 1‐related (HIP1R) has been recognized as a tumour suppressor in gastric cancer, while its biological function in PAAD remains to be elucidated. In this study, we reported the downregulation of HIP1R in PAAD tissues and cell lines, and the overexpression of HIP1R suppressed the proliferation, migration and invasion of PAAD cells, while silencing HIP1R showed the opposite effects. DNA methylation analysis revealed that the promoter region of HIP1R was heavily methylated in PAAD cell lines when compared to the normal pancreatic duct epithelial cells. A DNA methylation inhibitor 5‐AZA increased the expression of HIP1R in PAAD cells. 5‐AZA treatment also inhibited the proliferation, migration and invasion, and induced apoptosis in PAAD cell lines, which could be attenuated by HIP1R silencing. We further demonstrated that HIP1R was negatively regulated by miR‐92a‐3p, which modulates the malignant phenotype of PAAD cells in vitro and the tumorigenesis in vivo*.* The miR‐92a‐3p/HIP1R axis could regulate PI3K/AKT pathway in PAAD cells. Taken together, our data suggest that targeting DNA methylation and miR‐92a‐3p‐mediated repression of HIP1R could serve as novel therapeutic strategies for PAAD treatment.

## 1 INTRODUCTION

Pancreatic cancer (PAAD) is a highly malignant pancreatic adenocarcinoma which is characterized of high rate of metastasis, rapid development of drug resistance, high mortality and poor prognosis.^1^, ^2^ Patients with PAAD usually show no specific symptoms in early stage, posing a critical challenge for early diagnosis. Due to the aggressiveness of the diagnosed PAAD in advanced stage, the mortality rate of PADD is high and the 5‐year survival rate is only about 7%.^1^, ^2^ The choice of therapeutic strategies for PADD mainly depends on the stage of diagnosis, and the commonly used treatment approaches include surgical resection, chemotherapy, radiotherapy, interventional therapy and targeted therapy.^3^, ^4^ A considerable number of patients with early PADD diagnosis can be cured by surgical resection, while the treatment outcome for advanced patients with metastasis or drug resistance remains dismal.^5^ Understanding the molecular mechanisms underlying the progression of PADD is the key to providing insights into the formulation of novel targeted therapy in advanced stage.

The majority of current studies investigated the upregulated genes and their contributions to the progression of PAAD. For example, epiregulin is reported to be upregulated in pancreatic cancer to stimulate cell growth^.^ ^6^ The upregulation of telomerase in pancreatic cancer cells could support its resistance to etoposide treatment,^7^ and VEGF‐C overexpression is modulated by circNFIB1 to support the metastasis of PAAD cells.^8^ On the other hand, tumour suppressor genes represent important barriers for tumorigenesis and cancer progression, which are frequently mutated or down‐regulated in cancer cells.^9^, ^10^ Functional restoration of tumour suppressor genes are also attractive strategies to curb the progression of cancer.^10^ However, there is a lack of characterization of tumour suppressor genes in PAAD.

A recent study reported that Huntingtin‐interacting protein 1‐related (HIP1R) functions as a tumour suppressor in gastric cancer by inducing apoptosis and suppressing migration and invasion through targeting Akt.^11^ HIP1R has been recognized as a key component of clathrin‐coated pits and vesicles which links the endocytic machinery to the actin cytoskeleton.^12^ It also binds to 3‐phosphoinositides via ENTH domain to regulate signalling transduction and promotes cell survival by stabilizing receptor tyrosine kinases following ligand‐induced endocytosis.^13^ In colorectal cancer, HIP1R was reported to alter T cell‐dependent cytotoxicity by facilitating the lysosomal degradation of PD‐L1, which assists in the escape of immune surveillance of tumour cells.^14^ However, the biological functions of HIP1R in the progression of PAAD remain to be explored.

DNA methylation is an important epigenetic modification governing gene expression, which can be passed from parental cells to the next generation during DNA replication through the action of DNA methyltransferase.^15^, ^16^ The expression of oncogenes or tumour suppressor genes could be dysregulated by abnormal DNA methylation pattern. Aberrant DNA methylation has been implicated in cancer biology and is closely related to the occurrence and the progression of different types of cancer.^17^, ^18^ In addition, non‐coding RNAs such as microRNAs (miRNAs) add another layer of post‐transcriptional control on gene expression. miRNAs play regulatory roles in gene expression by targeting the complementary mRNA for degradation or arresting the translation.^19^ The deregulation of miRNAs contributes to the progression of cancer by modulating the expression of oncogene target or silencing tumour suppressor genes.^20^, ^21^ As a typical example, miR‐92a‐3p has been recognized as an oncogenic factor and its overexpression supports the uncontrolled proliferation and survival of tumour cells,^22^, ^23^ which has been proposed as an anticancer target for leukaemia and colorectal cancer.

In this study, we showed the downregulation of HIP1R in PAAD tissues and cell lines, and demonstrated that HIP1R serves as a tumour suppressor in PAAD since the overexpression of HIP1R suppressed the proliferation, migration and invasion, and induced apoptosis in PAAD cells. The promoter region of HIP1R was heavily methylated in PAAD cell lines, and the treatment of a DNA methylation inhibitor 5‐AZA increased HIP1R expression and impaired the malignant phenotype of PAAD cells. We further demonstrated that HIP1R was negatively regulated by miR‐92a‐3p, which modulates the malignant phenotype of PAAD cells in vitro and the tumorigenesis in vivo by targeting PI3K/AKT pathway. Taken together, our data showed that both DNA methylation and miR‐92a‐3p‐mediated repression of HIP1R contribute to the malignant progression of pancreatic cancer. Targeting DNA methylation and miR‐92a‐3p could serve as novel therapeutic strategies for PAAD treatment.

## 2 MATERIALS AND METHODS

### 2.1 Clinical samples

The tumour and para‐tumour tissue specimens were obtained from 106 PAAD patients at Tongji Hospital, Tongji Medical College, Huazhong University of Science and Technology. All experimental procedures were approved by the Medical Ethics Committee of Tongji Hospital, Tongji Medical College, Huazhong University of Science and Technology. All patients had signed the informed consent. The collected tissues were snap‐frozen in liquid nitrogen and stored at −80°C until further analysis.

### 2.2 Cell culture and 5‐AZA treatment

PAAD cell lines including PANC‐1, SW1990, BXPC‐3, AspC‐1 and normal pancreatic duct epithelial cell line HPDE‐6, and 293 T cell line were purchased from Shanghai Institutes for Biological Sciences, Chinese Academy of Sciences. Cells were cultured in DMEM (HyClone) complete medium containing 10% fetal bovine serum (Gibco) bovine serum, 100 μg/ml penicillin, 100 μg/ml streptomycin and 2 mmol/L glutamine in an incubator at 37°C with 5% CO2. 1 μM 5‐AZA (Sigma) was added in the cells to inhibit the DNA methyltransferase for 7 days, and the cells were collected for DNA and total RNA extraction.

### 2.3 Methylation‐specific PCR (MSP) and bisulphite sequencing PCR (BSP)

QIAamp DNA Mini Kit (Qiagen) was used to extract genomic DNA from cells. DNA was denatured by adding NaOH for 10 min at 37°C and then mixed with freshly prepared sodium bisulphite mixture (Qiagen). After bisulphite modification at 50°C for 16 h, the DNA sample was purified by ethanol precipitation and re‐dissolved in solution with 10 mM Tris/0.1 mM EDTA. PCR reaction was performed to amplify the bisulphite‐treated DNA using the following program: 95°C for 5 min, followed by 35 cycles of 94°C for 35 s, 60°C for 35 s, and 72°C for 35 s. Finally, the methylated primers were substituted by unmethylated primers to perform PCR amplification (using an annealing temperature of 58°C for 35 cycles). For BSP analysis, the EZ DNA Methylation‐Gold™ Kit (ZYMO RESEARCH) was used in this study according to the manufacturer's instructions.

### 2.4 Real‐time RT‐PCR

Total RNA from the tissues and cells was extracted using MiniBEST Universal RNA Extraction Kit (TaKaRa) according to the instruction manual. The extracted total RNA was dissolved in DEPC water, and its concentration was measured with NanoDrop. 5 μg of total RNA was used for reverse‐transcription into cDNA using PrimeScript™ RT reagent kit (TaKaRa) The relative expression of HIP1R, DNMT1, DNMT3A, DNMT3B and miR‐92a‐3p was determined by SYBR Premix EX Taq™ kit (TaKaRa) in a 7500 Real‐Time PCR System (Applied Biosystems). The following cycling condition was used for qPCR: 95°C for 30 s, followed by 40 cycles of 95°C for 5 s, and 60°C for 10 s. The relative expression level was quantified by ${{2^{-}}^{\Delta \Delta }}^{C_{t}}$ approach with the internal control of GAPDH and U6 snRNA gene.

Primers used for qPCR were synthesized by Sangon Biotechnology Co., Ltd. (Shanghai, China):

HIP1R:

F‐ CGAGCAGTTCGACAAGACC; R‐ GTGTGCCCAGAATGATGCG.

DNMT1:

F‐ CCTAGCCCCAGGATTACAAGG; R‐ ACTCATCCGATTTGGCTCTTTC.

DNMT3A:

F‐ CCGATGCTGGGGACAAGAAT; R‐ CCCGTCATCCACCAAGACAC.

DNMT3B:

F‐ AGGGAAGACTCGATCCTCGTC; R‐ GTGTGTAGCTTAGCAGACTGG.

miR‐92a‐3p:

F‐ GCAATCATGTGTATAGATATG; R‐ CTCCACTCACAGAGGTGTC.

GAPDH:

F‐ CTGGGCTACACTGAGCACC; R‐ AGTGGTCGTTGAGGGCAATG.

U6:

F‐ TGCGGGTGCTCGCTTCGGCAGC; R‐ CCAGTGCAGGGTCCGAGGT.

### 2.5 Plasmid constructions and transfection

The miR‐92a‐3p mimic and miR‐92a‐3p inhibitor and the corresponding controls were purchased from General Biosystem (General Biosystem).^24^ The cDNA of HIP1R was cloned into pcDNA3.1 plasmid for overexpressing HIP1R, and siRNA targeting HIP1R and scramble control siRNA were produced by Guangzhou RiboBio. All these above molecules were transfected into cells using Lipofectamine 2000 (Thermo Fisher Scientific). Briefly, 60% confluent cells in 6‐well plate were transfected with 50 nM of microRNA mimic or inhibitor or 6 μg of pcDNA3.1‐HIP1R plasmid according to manufacturer's instruction. Transfected cells were subjected to subsequent analysis 48 h post‐transfection.

### 2.6 CCK‐8 proliferation assay

Cell proliferation was examined by Cell Counting Kit‐8 (CCK8, Dojindo Laboratory) according to manufacturer's instructions. 48 h after transfection, cells were seeded into a 96‐well plate at a density of 1500 cell/well and cultured for 0, 24, 48, 72 and 96 h, respectively. Subsequently, 10 μl CCK8 reaction solution was added to each well at indicated time point and incubated for 1 h. The light absorption value (OD value) in each condition was captured at 450 nm wavelength on a Synergy H1 microplate reader.

### 2.7 Colony formation assay

Cells were trypsinized and seeded into a 6‐well plate at the density of 2000 cells/well. The culture medium was changed every 3 days for 2 weeks. Cells were then fixed with 4% paraformaldehyde (BD Biosciences) at room temperature for 10 min and stained with 0.5% crystal violet (Beyotime) for 20 min. The number of stained colonies was counted under an inverted microscope (Olympus).

### 2.8 Migration and invasion assay

Cells were trypsinized and re‐suspended in serum‐free medium. The transwell upper chamber (Corning) without Matrigel (BD Biosciences) was used for migration assay, while transwell upper chamber coated with Matrigel was used for invasion assay. 5 × 10^5^ cells were inoculated into the transwell upper chamber in serum‐free medium and 500 μl of 10% serum‐containing medium was added to the lower chamber. After 24 h, culture medium was discarded and the cells were fixed with 4% paraformaldehyde for 10 min and stained with 0.5% crystal violet (Beyotime) for 20 min. Cells were photographed under an inverted microscope (Olympus), and cells from five randomly selected fields in each sample were counted.

### 2.9 Cell apoptosis analysis

Annexin‐V fluorescein isothiocyanate/PI apoptosis detection kit (Invitrogen Life Technologies) was used for apoptosis detection. Briefly, cells with different treatments were trypsinized and re‐suspended in 1000 μl staining buffer with 1 million cells. 1 μl Annexin V‐FITC and 1 μl PI were added to the cell suspension for 30 min incubation in the dark. Stained cells were centrifuged and washed twice with 1xPBS and resuspended in 400 μl staining buffer. The BD FACS CantoTM II Flow Cytometer (BD Biosciences) was used to determine cell apoptotic populations.

### 2.10 Immunohistochemistry (IHC)

Immunohistostaining was performed on 5‐μm sections of formalin‐fixed paraffin‐embedded (FFPE) tissues. The sections were deparaffinized and hydrated by three washes of xylene for 5 min each, two washes of 100% ethanol for 10 min each, two washes of 95% ethanol for 10 min each, and in two washes in dH2O for 5 min each. After antigen retrieval with citrate buffer at 95°C for 10 min, the sections were washed in dH2O three times and then incubated in 3% hydrogen peroxide for 10 min. After three times washes in TBST buffer for 5 min, the section was blocked for 1 h using 10% goat serum (Sigma, Germany) at 37°C, followed by the incubation with primary antibodies (HIP1R: Abcam ab226197; Ki67: Abcam ab15580) overnight at 4°C. Next day, the slides were washed 3 times with PBS and incubated with SignalStain® Boost Detection Reagent (HRP Rabbit, Cell Signaling Technologies) and incubated in a humidified chamber for 30 min. The signal development was performed for 5 min using 400 μl SignalStain® substrate (Cell Signaling Technologies). The section was washed in dH2O two times and mounted with coverslips using the mounting medium (Cell Signaling Technologies) before imaging under an inverted microscope (Olympus). The IHC staining was examined and scored independently by two experienced pathologists.

### 2.11 Dual‐luciferase reporter assay

The HIP1R 3’‐UTR containing the wildtype binding site of miR‐92a‐3p (WT) or the mutated binding site (MUT) was cloned into the psiCHECK2 dual‐luciferase reporter vector (Promega, WI, USA). 293 T cells were transfected with WT or MUT luciferase reporter with either miR‐92a‐3p mimic or miR‐NC using Lipofectamine 3000 (Invitrogen). After 48 h, the relative luciferase activities were measured using Dual‐Luciferase Reporter Assay Kit (Promega) on a luminescence microplate reader (Infinite 200 PRO, Tecan). The relative firefly luciferase activity in the reporter plasmid was normalized to that of Renilla luciferase.

### 2.12 Western blot analysis

Total protein from cells and tissues was extracted by ice‐cold RIPA lysis buffer (Beyotime) containing 1% PMSF. Cells suspended in RIPA buffer were lysed on ice for 10 min and lysates were centrifuged at 13000 *g* for 10 min. The supernatant was quantified by a BCA Protein assay kit (Beyotime Biotechnology Shanghai, China). 10 μg of total protein was used for SDS‐PAGE electrophoresis and transferred onto the PVDF membrane (Bio‐Rad). After blocking with 5% skimmed milk for 1 h, the membrane was then incubated with primary antibodies: HIP1R (1:1000; Abcam ab226197), β‐Actin (1:2000, Abcam ab8227), Akt (1:1000, Abcam ab8805), p‐Akt (1:2000; Abcam ab18206), mTor (1:1000; Abcam ab2732), p‐mTor (1:1000; Abcam ab131538), S6K (1:1000; Abcam ab9366), p‐S6K (1:1000; Abcam ab131436) for overnight at 4°C. The membrane was washed 3 times with TBST buffer and incubated with HRP‐linked secondary antibody (1:3000; Cell Signaling Technologies). The protein bands were developed using an enhanced ECL chemiluminescence kit (Solarbio) and photographed on a gel imager system (Bio‐Rad). ImageJ software (Bethesda) was used for the densitometry analysis.

### 2.13 In vivo tumorigenesis model

Animal experiments were approved by the Institutional Animal Care and Use Committee of the Tongji Hospital, Tongji Medical College, Huazhong University of Science and Technology. 1 × 10^6^ SW1990 PAAD cells were subcutaneously injected into 18 nude mice to establish xenograft. The mice were divided into three groups: miR‐92a‐3p mimic injection, inhibitor miR‐92a‐3p injection and PBS injection (*n* = 6 in each group). All the injections were performed intravenously twice a week for 2 weeks until the tumour volume reached approximately 50 mm^3^. Tumour volume was continuously monitored for 24 days and all the mice were euthanized by CO2 asphyxiation and cervical dislocation. The xenograft tumours were removed and weighed, and the samples were subject to further analysis.

### 2.14 Bioinformatics analyses

UCSC Genome Database (http://genome.ucsc.edu) was used to locate the HIP1R gene locus and multiple CpG islands in the promoter region. To identify the potential microRNAs targeting HIP1R, miRWalk (http://mirwalk.umm.uni‐heidelberg.de/), miRTarbase (http://mirtarbase.mbc.nctu.edu.tw/index.html) and Targetscan (http://www.targetscan.org/vert_72/) online databases were used to search for the interacting miRNAs of HIP1R mRNA. To understand the gene programs regulated by HIP1R, GSEA (Gene Set Enrichment Analysis) was performed (http://www.gsea‐msigdb.org/gsea/index.jsp) to identify gene sets correlated with high or low HIP1R expression.

### 2.15 Statistical analysis

All data were analysed by SPSS 20.0 software (IBM Corp). The difference between two groups was analysed by Student's *t*‐test (two‐tailed), and one‐way anova was performed for comparisons among multiple groups, with Dunn's test for correction of multiple comparisons. Spearman correlation analysis was employed to determine the relationship between HIP1R and miR‐92a‐3p expression levels. The overall survival rate was analysed by Kaplan–Meier Survival curve and log‐rank test. Data were displayed as mean ± standard deviation (x ± s) of at least 3 independent experiments. *p* < 0.05 was considered as statistically significant.

## 3 RESULTS

### 3.1 HIP1R expression is significantly downregulated in PAAD tissues

**FIGURE 1:**
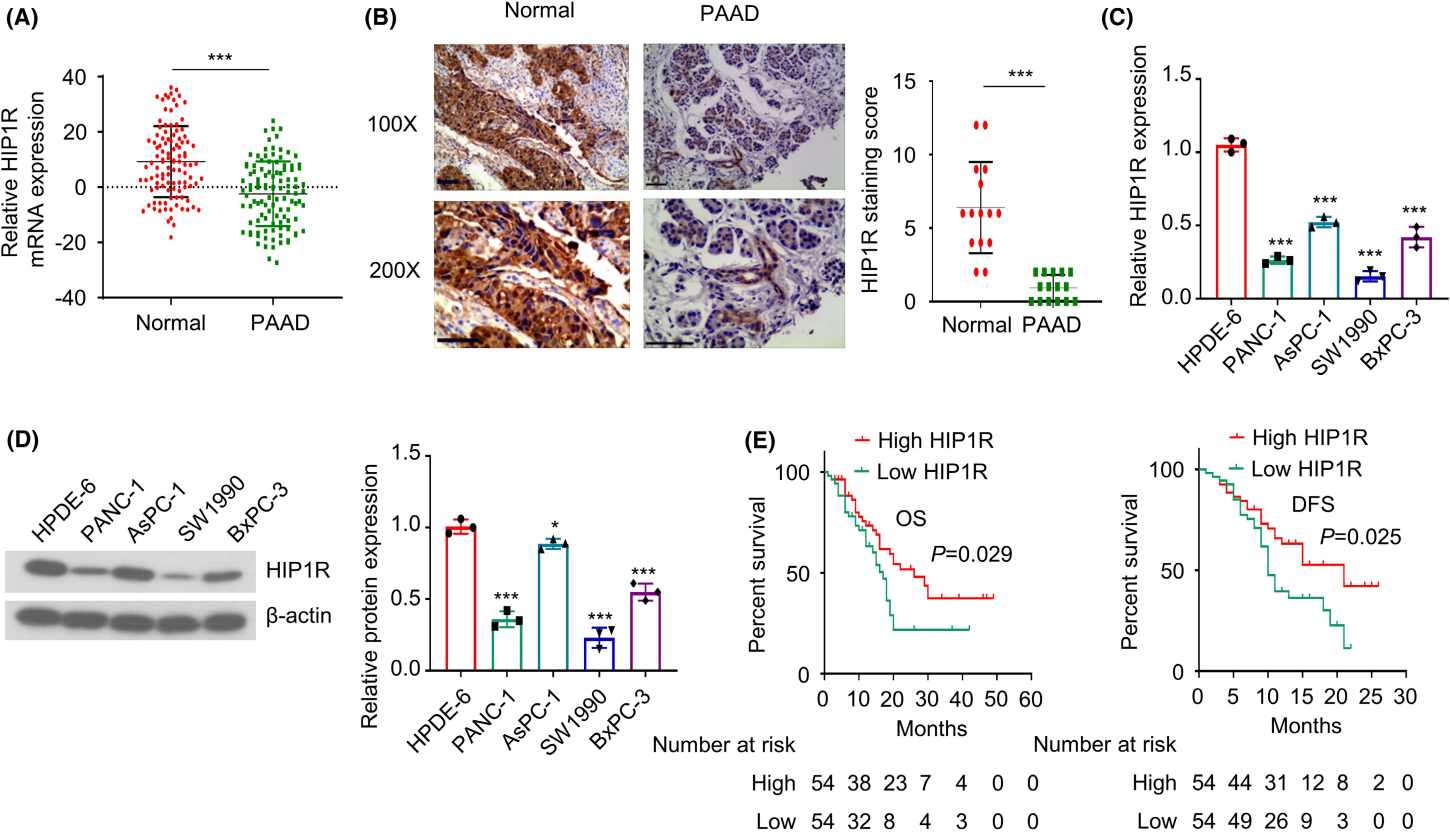
HIP1R is significantly downregulated in PAAD tumour tissue and cells. (A) The relative mRNA expression levels of HIP1R in PAAD tumour tissues and the matched para‐cancerous normal tissues of 106 patients were examined by qRT‐PCR. (B) The protein level of HIP1R in PAAD tumour tissues and the matched para‐cancerous normal tissues were analysed in 20 randomly selected PAAD patients. Images showed representative IHC images of HIP1R (scale bar: 50 μm). (C) The mRNA levels of HIP1R were quantified in PAAD cell lines and pancreatic ductal epithelial cell line by qRT‐PCR (D) Western blot analysis of HIP1R expression in PAAD cell lines and pancreatic ductal epithelial cell line. (E) Kaplan–Meier curve analysis of the overall survival (OS) and disease‐free survival (DFS) in HIP1R high and low‐expression PAAD patients. *p < 0.05, **p < 0.01, ***p < 0.001

To explore the potential role of HIP1R in PAAD, the mRNA and protein levels of HIP1R were compared between PAAD tumour tissues (*n* = 106) and the matched pericarcinomatous tissues (*n* = 106) by qRT‐PCR and IHC. The results showed that compared with the adjacent normal tissues, HIP1R level was significantly lower in PAAD tissues (Figure 1A,B and Figure S1). We also evaluated the mRNA and protein levels of HIP1R in PAAD cell lines (PANC‐1, SW1990, BXPC‐3 and AspC‐1) and normal pancreatic duct epithelial cell line HPDE‐6. The results also showed that HIP1R expression level was significantly reduced in PAAD cell lines (Figure 1C,D). To evaluate the association between HIP1R and the survival of PAAD patients, the PAAD patients were divided into HIP1R low‐expression group (*n* = 53) and high‐expression group (*n* = 53) according to median expression level of HIP1R measured by qRT‐PCR, and their clinicopathological characteristics were summarized in Table 1. Chi‐square test showed that low HIP1R expression was significantly correlated with more advanced TNM stages, larger tumour sizes and more lymph node metastasis (Table 1). Furthermore, Kaplan–Meier curve analysis revealed that low HIP1R expression was associated with a poorer overall survival (OS) and disease‐free survival (DFS) in PAAD patients (Figure 1E). Together, these data indicate that HIP1R may act as a tumour suppressor which is downregulated in PAAD tissues and cells.

**TABLE 1:** Correlations of HIP1R expression with clinicopathologic features of pancreatic cancer

Factor	HIP1R expression (Cut‐off: median value of qRT‐PCR expression)		*p* Value
	Low (*n* = 53)	High (*n* = 53)	
Age			
≤50	35	28	0.166
>50	18	25	
Gender			
Female	17	24	0.163
Male	36	29	
Tumor size			
≤2 cm	21	34	0.012
>2 cm	32	19	
TNM stage			
I/II	14	27	0.01
III/IV	39	26	
Lymph node metastasis			
Negative	23	35	0.019
Positive	30	18	

### 3.2 HIP1R acts as a tumour suppressor in the regulation of proliferation, migration and invasion and the survival of PAAD cells

**FIGURE 2:**
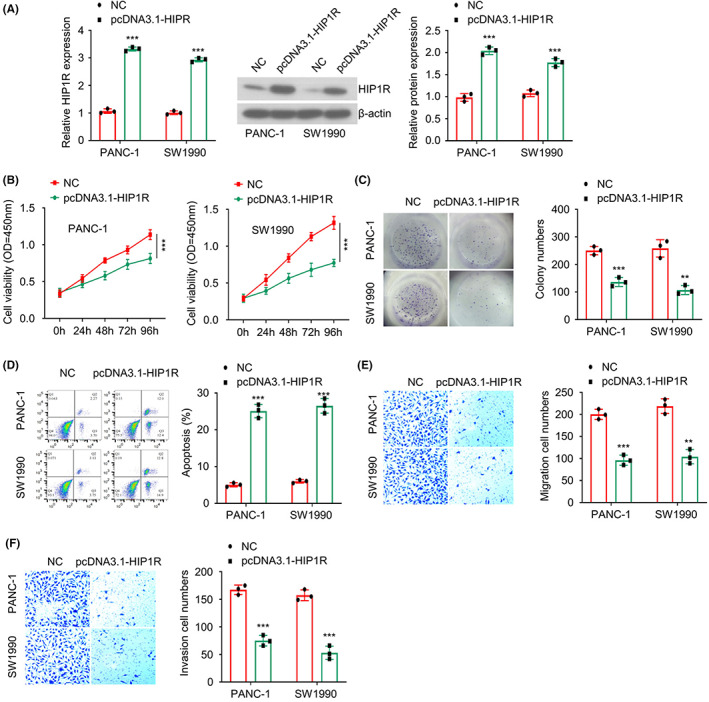
The overexpression of HIP1R suppressed cell proliferation, migration and invasion and induced apoptosis. (A) The mRNA and protein levels of HIP1R in PANC‐1 and SW1990 cells were examined after the transfection of pcDNA3.1‐HIP1R expression vector or empty vector (NC). (B) Cell proliferation of PAAD cells in different groups (NC group and pcDNA3.1‐HIP1R group) was assessed by CCK8 assay. (C) Clonogenic abilities of PAAD cells in different groups (NC group and pcDNA3.1‐HIP1R group) were determined by colony formation assay. (D) The percentage of apoptotic events of PAAD cells in different groups (NC group and pcDNA3.1‐HIP1R group) was analysed by flow cytometry. (E, F) Cell migration and invasion abilities of PAAD cells in different groups (NC group and pcDNA3.1‐HIP1R group) were examined by Transwell migration and invasion assays. *p < 0.05, **p < 0.01, ***p < 0.001

To validate the tumour suppressor role of HIP1R, we constructed pcDNA3.1‐HIP1R expression vector to overexpress HIP1R in PAAD cells. As compared to the cells transfected empty vector (NC), pcDNA3.1‐HIP1R transfection significantly increased the mRNA and protein level of HIP1R in PANC‐1 and SW1990 cells (Figure 2A). CCK‐8 proliferation assay and colony formation assay demonstrated that HIP1R overexpression suppressed cell proliferation and colony formation ability in PAAD cells (Figure 2B,C). In contrast, HIP1R overexpression induced more apoptotic events in both cell lines (Figure 2D). The transwell migration and invasion assay also revealed that HIP1R overexpression could impair the abilities of cell migration and invasion (Figure 2E,F).

We also applied siRNA targeting HIP1R to reduce the expression of HIP1R in PAAD cells (Figure S2A,B). Upon HIP1R knockdown, PANC‐1 and SW1990 cells showed augmented cell proliferation (Figure S2C), enhanced colony formation ability (Figure S2D) and stronger migratory and invasive capabilities (Figure S2E,F). Taken together, these data indicate that HIP1R functions as a tumour suppressor to negatively regulate the malignancy of PAAD cells.

### 3.3 PAAD downregulation is mediated by DNA hypermethylation in PAAD

**FIGURE 3:**
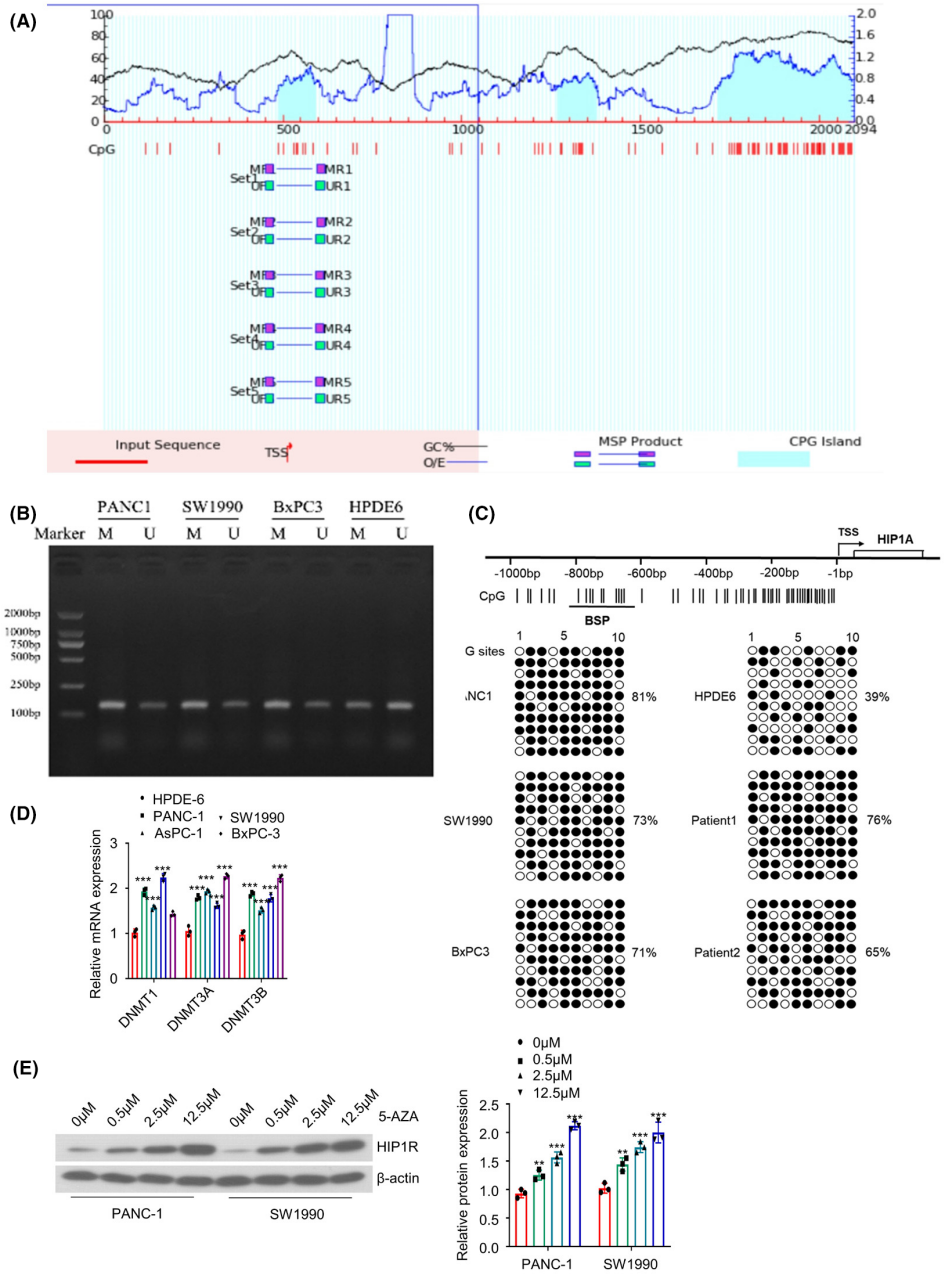
DNA hypermethylation mediates the downregulation of HIP1R. (A) The CpG islands in the promoter region of HIP1R gene were predicted by UCSC genome browser. (B) Methylation‐specific PCR (MSP) was performed to compare the methylation level at the CpG islands between PAAD cell lines (PANC‐1, SW1990 and BxPC3) and normal pancreatic duct epithelial cell line HPDE‐6. (C) Bisulphite sequencing PCR (BSP) was employed to profile the methylation levels of HIP1R promoter region in PAAD cell lines, HPDE‐6 cell line and PAAD tissues. (D) The relative mRNA levels of DNMT1，DNMT3A，DNMT3B were quantified by qRT‐PCR. (E) The mRNA and protein levels of HIP1R were examined by qRT‐PCR and Western blot after the treatment of different doses of 5‐AZA. *p < 0.05, **p < 0.01, ***p < 0.001

To understand the mechanism underlying HIP1R downregulation, UCSC Genome Database (http://genome.ucsc.edu) was used to locate the HIP1R gene and multiple CpG islands in the promoter region (Figure 3A). We then performed methylation‐specific PCR (MSP) to compare the methylation level at the CpG islands between PAAD cell lines and normal pancreatic duct epithelial cell line HPDE‐6, which showed an elevated methylation level in PAAD cell lines (PANC‐1, SW1990 and BxPC3) (Figure 3B). We also conducted bisulphite sequencing PCR (BSP) in PAAD cell lines, HPDE‐6 cell line and PAAD tissues. The methylation levels of HIP1R promoter in PAAD cell lines (PANC‐1: 81%, SW1990: 73%, BxPC‐3: 71%) and PAAD patient tissues (76% and 65%) were much higher than that of normal pancreatic duct epithelial cell line HPDE‐6 (39%) (Figure 3C). Additionally, qRT‐PCR analysis revealed that multiple DNA methyltransferases, including DNMT1, DNMT3A and DNMT3B, were considerably upregulated in PADD cells than in normal pancreatic duct epithelial cells (Figure 3D). To confirm that the downregulation of HIP1R was dependent on DNA methylation, we treated PAAD cells with 5‐AZA (a DNA methylation inhibitor) for 48 h. The inhibition of DNA methylation increased HIP1R in a dose‐dependent manner (Figure 3E). Together, these data suggest PAAD downregulation is mediated by DNA hypermethylation in PAAD tissues and cell lines.

### 3.4 5‐AZA treatment impairs the malignancy of PAAD cells in a HIP1R‐dependent manner

**FIGURE 4:**
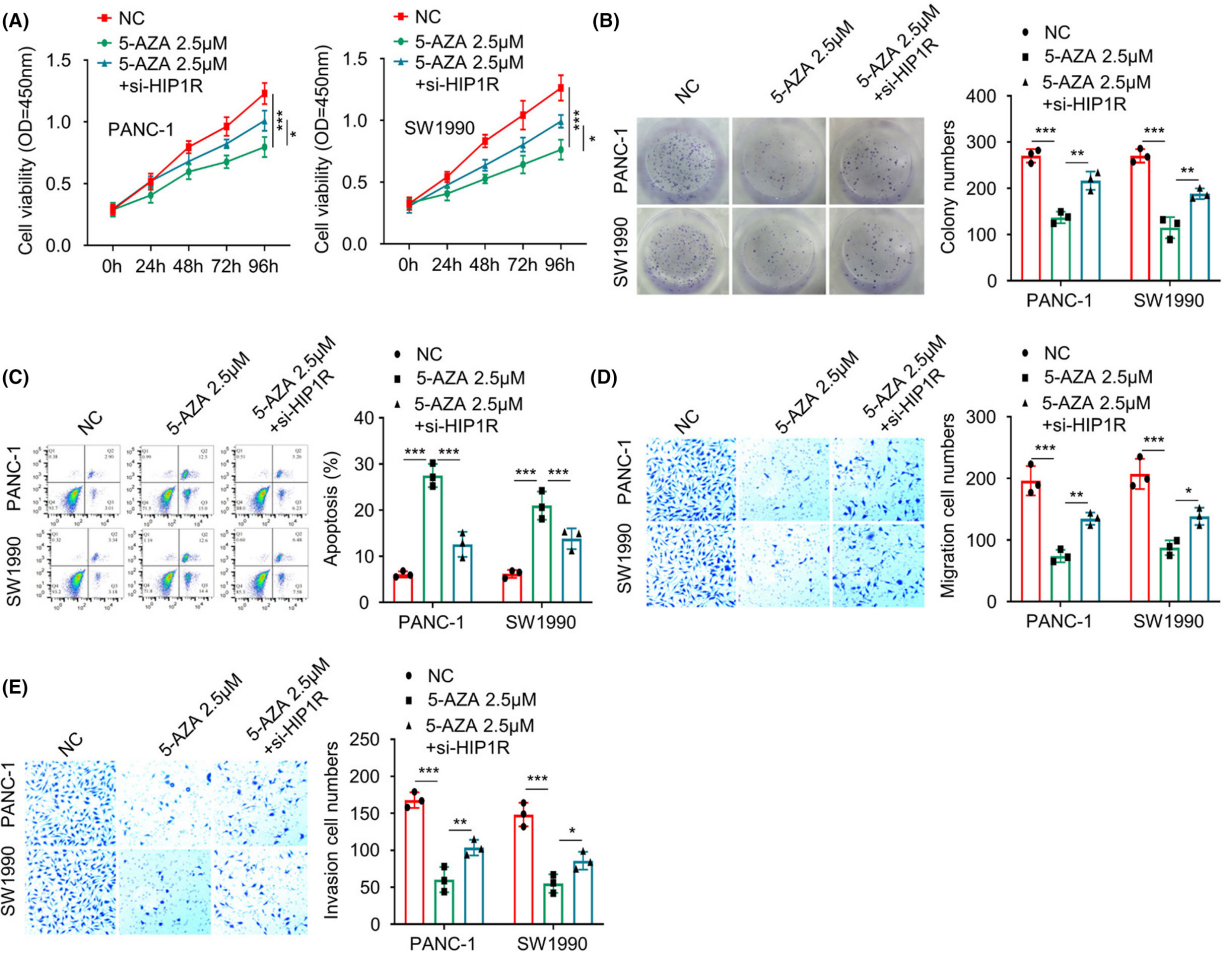
5‐AZA treatment impairs the malignancy of PAAD cells in a HIP1R‐dependent manner. (A) Cell proliferation abilities of PAAD cells in different groups (NC group, 5‐AZA group, 5‐AZA + HIP1R‐siRNA group) were examined by CCK8 assay (B) Clonogenic abilities of PAAD cells in different groups (NC group, 5‐AZA group, 5‐AZA + HIP1R‐siRNA group) were determined by colony formation assay. (C) The apoptotic events of PAAD cells in different groups (NC group, 5‐AZA group, 5‐AZA + HIP1R‐siRNA group) were quantified by flow cytometry. (D, E) Cell migration and invasion abilities of PAAD cells in different groups (NC group, 5‐AZA group, 5‐AZA + HIP1R‐siRNA group) were assessed by Transwell assays. *p < 0.05, **p < 0.01, ***p < 0.001

To investigate the impact of DNA hypermethylation on the malignant phenotype, PANC‐1 and SW1990 cells were treated with 2.5 μM 5‐AZA for 48 h with or without HIP1R silencing by siRNA. We observed that compared to the control (NC), 5‐AZA treatment significantly impaired the malignant phenotypes including cell proliferation, clonogenic ability, cell migration and invasion and induced apoptosis. (Figure 4A–E). The co‐transfection of si‐HIP1R largely attenuated the effect of 5‐AZA (Figure 4A–E). Therefore, DNA hypermethylation contributes to the malignant phenotype by modulating HIP1R expression.

### 3.5 miR‐92a‐3p overexpression contributes to the downregulation of HIP1R in PAAD tissue and cells

**FIGURE 5:**
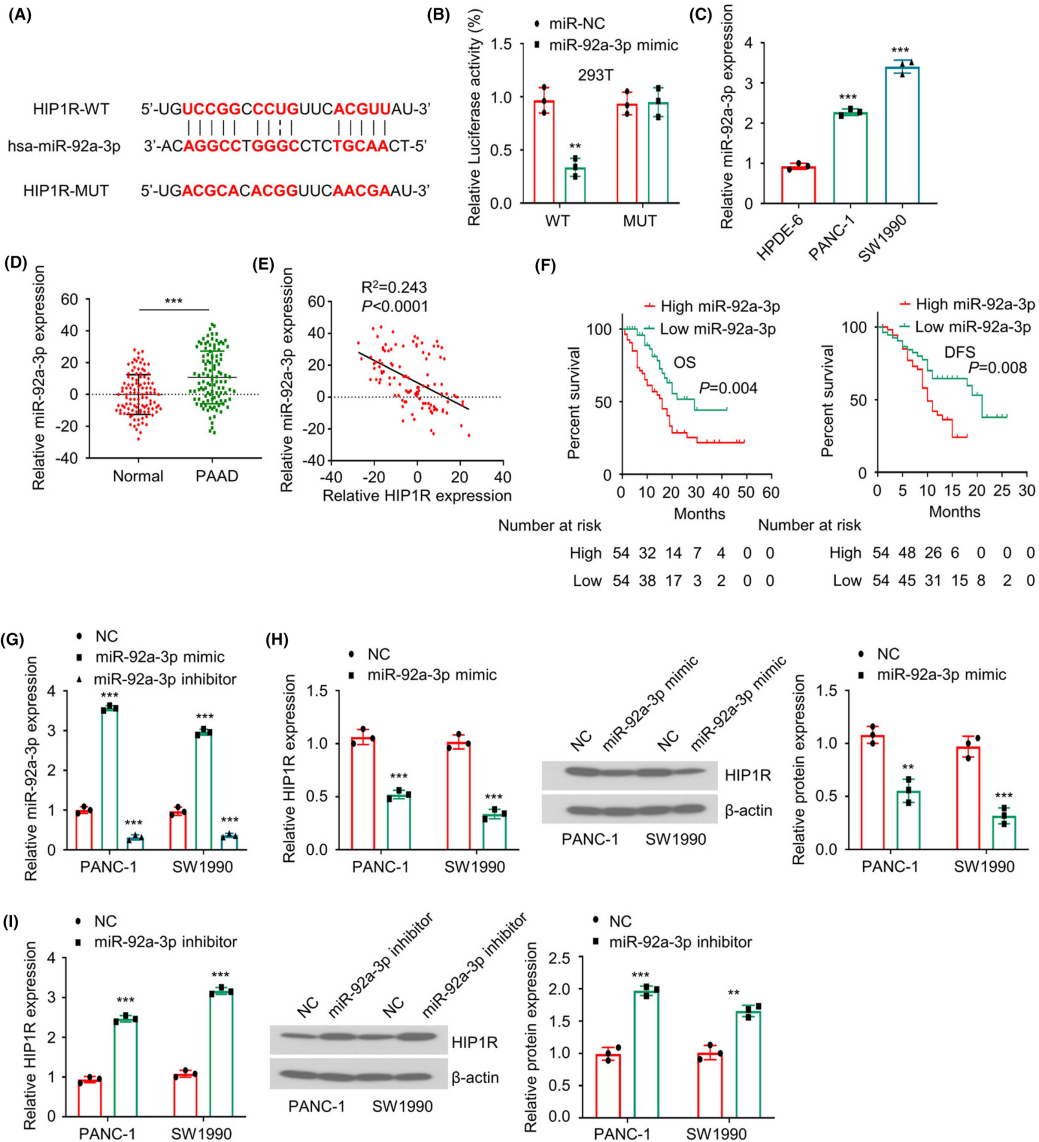
miR‐92a‐3p regulates the expression of HIP1R in PAAD tissue and cells (A) The predicted miR‐92a‐3p binding sites on the 3' UTR of HIP1R mRNA. (B) Dual‐luciferase reporter assay was performed using WT (wildtype binding sites) or MUT (mutated binding sites) luciferase reporter in the presence of miR‐92a‐3p mimic or miR‐NC. (C) The relative miR‐92a‐3p levels in PAAD cell lines and pancreatic ductal epithelial cell line were determined by qRT‐PCR. (D) The relative miR‐92a‐3p levels in PAAD tumour tissues and matched adjacent tissues were determined by qRT‐PCR. (E) Spearman correlation analysis shows a negative correlation between HIP1R mRNA level and miR‐92a‐3p expression level. (F) High miR‐92a‐3p expression in tumour tissues was associated with a poorer overall and disease‐free survival of PAAD patients, as revealed by Kaplan–Meier method. (G) The expression levels of miR‐92a‐3p were analysed by qRT‐PCR in PANC‐1 and SW1990 cells after the transfection of miR‐92a‐3p mimic or inhibitor. (H, I) The mRNA and protein levels of HIP1R in PANC‐1 and SW1990 cells were examined by qRT‐PCR and Western blot after the transfection of miR‐92a‐3p mimic or inhibitor. *p < 0.05, **p < 0.01, ***p < 0.001

To explore whether non‐coding RNAs such as microRNAs also mediates the downregulation of HIP1R, miRWalk (http://mirwalk.umm.uni‐heidelberg.de/), miRTarbase (http://mirtarbase.mbc.nctu.edu.tw/index.html) and Targetscan (http://www.targetscan.org/vert_72/) online databases were used to search for the interacting miRNAs of HIP1R mRNA. It was found that there were potential binding sites between miR‐92a‐3p and the 3' UTR (untranslated region) of HIP1R mRNA (Figure 5A). To validate the functional interaction, the HIP1R 3' UTR containing the wildtype binding site of miR‐92a‐3p (WT) or the mutated binding site (MUT) was cloned into the dual‐luciferase reporter vector, and 293 T cells were transfected with WT or MUT luciferase reporter with either miR‐92a‐3p mimic or miR‐NC. miR‐92a‐3p mimic significantly decreased the luciferase activity of the WT reporter, while the effect was abrogated when the binding sites were mutated (Figure 5B). qRT‐PCR analysis further showed that miR‐92a‐3p level was significantly upregulated both in PAAD tissues and cells, when compared to normal tissues and cell line (Figure 5C,D). Besides, the Spearman coefficient test revealed a negative correlation between miR‐92a‐3p expression and HIP1R level in PAAD tissues (Figure 5E), which suggests that the miR‐92a‐3p upregulation may control HIP1R expression in PAAD cells and tissues. Furthermore, the Kaplan–Meier curve analysis demonstrated that PAAD patients with high miR‐92a‐3p expression were associated with a poorer prognosis in both overall survival and disease‐free survival (Figure 5F).

To investigate the effect of miR‐92a‐3p on HIP1R expression, miR‐92a‐3p mimic or miR‐92a‐3p inhibitor was transfected into PANC‐1 and SW1990 cells to increase or decrease miR‐92a‐3p expression (Figure 5G). Upon the transfection of miR‐92a‐3p mimic, HIP1R expression was significantly reduced at both mRNA and protein level (Figure 5H), while miR‐92a‐3p inhibition increased HIP1R expression (Figure 5I). These data indicate that miR‐92a‐3p overexpression contributes to the downregulation of HIP1R in PAAD tissue and cells. However, when cells were treated with 5‐AZA, the expression level of miR‐92a‐3p was not affected, which suggests that the expression of miR‐92a‐3p is not regulated by DNA methylation (Figure S3).

### 3.6 miR‐92a‐3p regulates the phenotypes of PAAD cells in a HIP1R‐dependent manner

To investigate whether miR‐92a‐3p regulates the phenotype of PAAD phenotype, we transfected PANC‐1 and SW1990 cells with miR‐92a‐3p mimic. Compared to the cells transfected with miR‐NC control, the cell proliferation, colony formation ability, cell migration and invasion were significantly augmented and the apoptotic events were reduced in cells transfected with miR‐92a‐3p mimic (Figure S4A‐E). When HIP1R‐pcDNA3.1 was co‐transfected, the effects of miR‐92a‐3p mimic were partially suppressed (Figure S4A‐E). These results indicate that miR‐92a‐3p regulates HIP1R expression to modulate the phenotypes of PAAD cells.

### 3.7 miR‐92a‐3p regulates tumorigenesis and HIP1R expression in the xenograft model of PAAD cells

**FIGURE 6:**
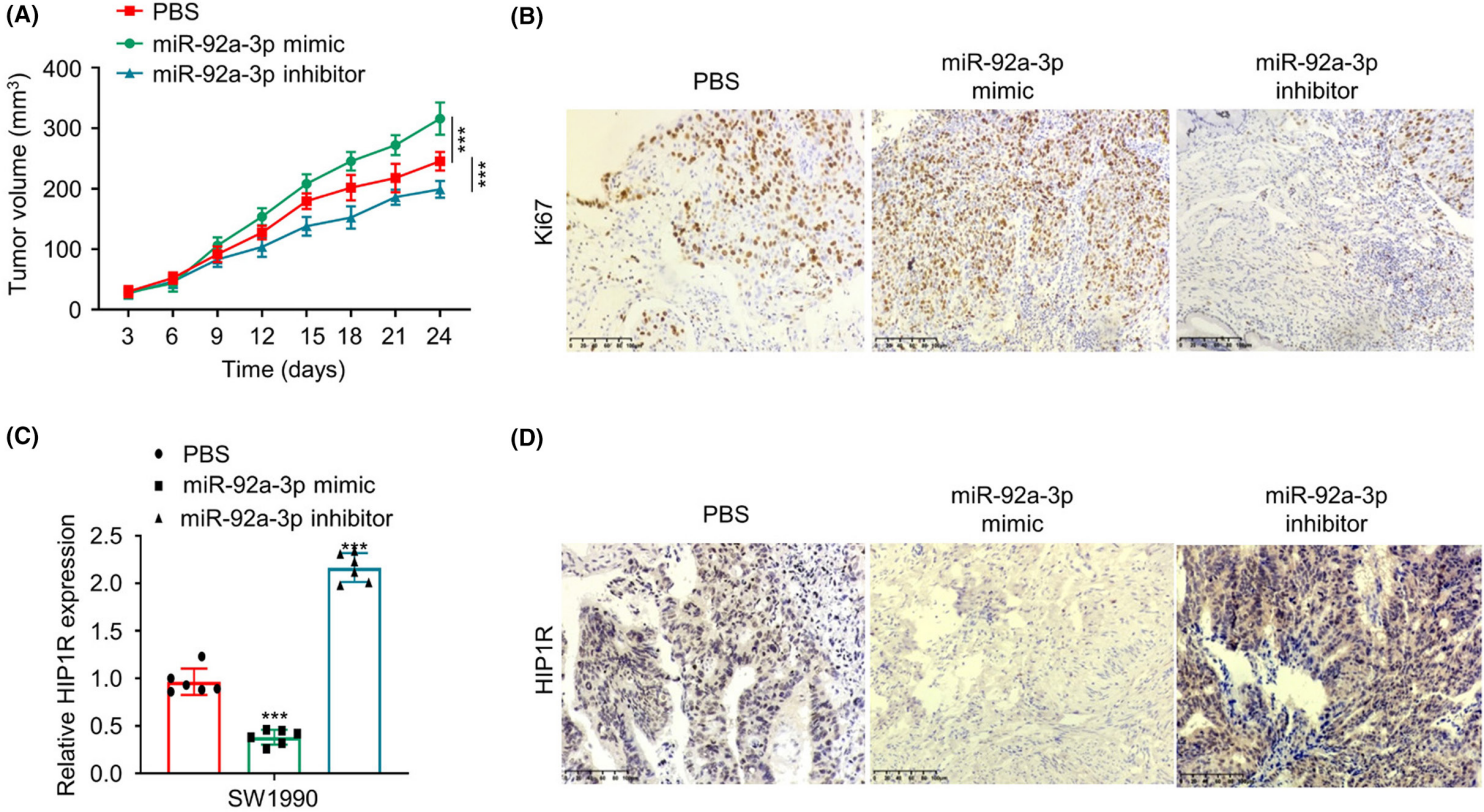
miR‐92a‐3p regulates tumorigenesis and HIP1R expression in the xenograft model of PAAD cells. (A) The tumour volume was monitored for 24 days in nude mice after the injection of PBS, miR‐92a‐3p mimic or inhibitor. (B) The protein level of Ki67 in the xenograft tissues was measured by IHC staining. (C) The mRNA levels of HIP1R in the xenograft tissues were determined by qRT‐PCR from nude mice after the injection of PBS, miR‐92a‐3p mimic or inhibitor. (D) The protein level of HIP1R in the xenograft tissues was measured by IHC staining. ***p < 0.001

To further study the role of miR‐92a‐3p in the tumorigenesis, SW1990 cells were subcutaneously injected into nude mice to establish xenograft, and the mice were divided into three groups: miR‐92a‐3p mimic injection, inhibitor miR‐92a‐3p injection and PBS injection. The results showed that miR‐92a‐3p mimic promoted tumour growth while the inhibition of miR‐92a‐3p impaired the tumour volume (Figure 6A). Consistently, IHC staining of the cell proliferation marker Ki67 demonstrated that miR‐92a‐3p mimic increased the number of Ki67 expressing cells, while miR‐92a‐3p inhibitor reduced the number of cells stained positive for Ki67 (Figure 6B). We also quantified the expression of HIP1R in the xenograft samples using qRT‐PCR and IHC staining. As expected, the mRNA level and protein level of HIP1R were decreased in the xenograft samples with miR‐92a‐3p mimic injection, and miR‐92a‐3p inhibitor increased HIP1R expression (Figure 6C,D). These results further corroborate the functional role of miR‐92a‐3p in the tumorigenesis of PAAD.

### 3.8 Overexpression of HIP1R or inhibition of miR‐92a‐3p modulates the malignancy of PAAD cells by targeting PI3K/Akt pathway

**FIGURE 7:**
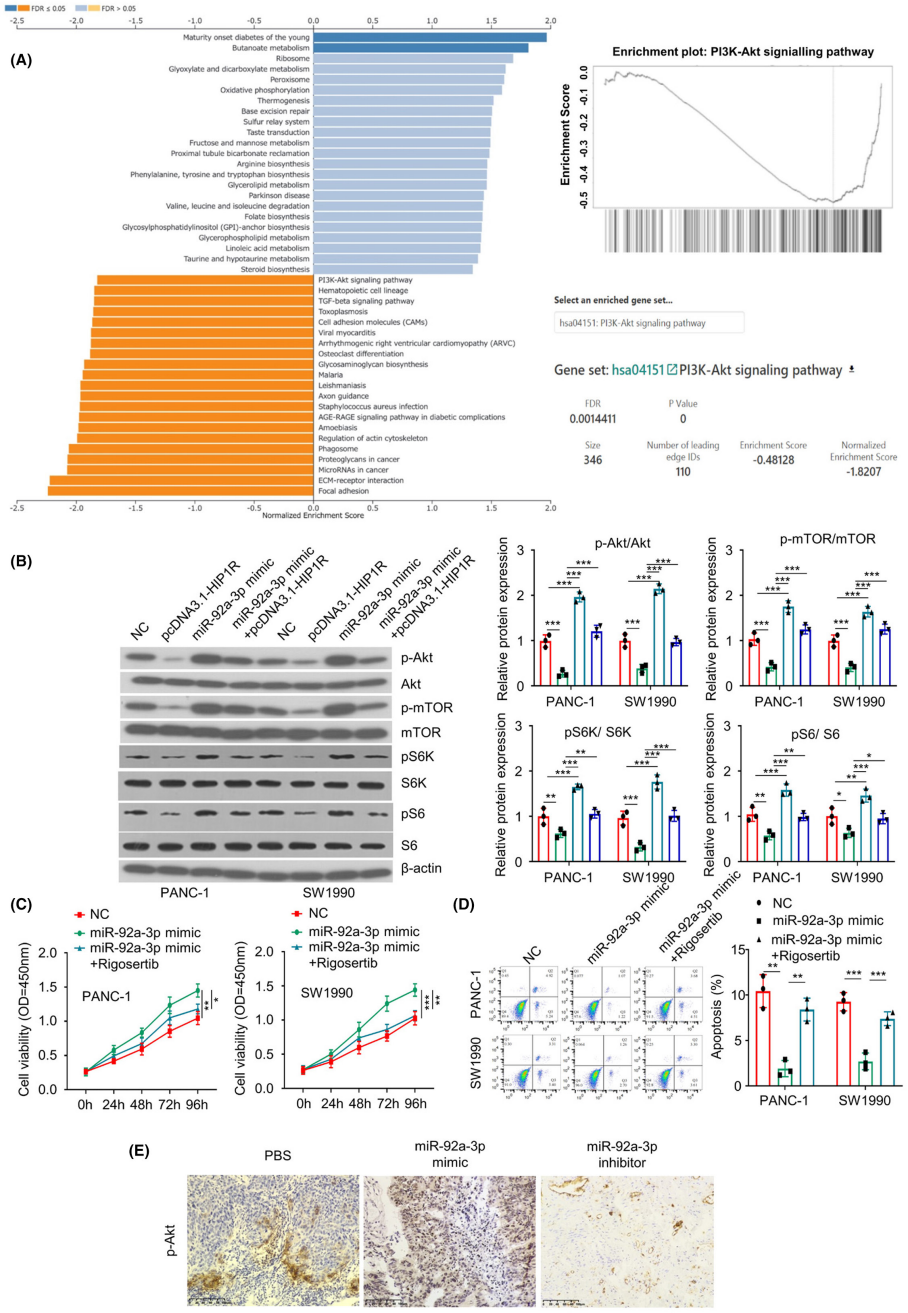
Overexpression of HIP1R or inhibition of miR‐92a‐3p modulates the malignancy of PAAD cells by targeting PI3K/Akt pathway. (A) GSEA analysis revealed a negative correlation between HIP1R expression and the gene expressions in PI3K/Akt pathway. (B) The phosphorylation levels of Akt, mTOR, S6K and S6 were examined by Western blot in PAAD cells with different treatments (NC group, pcDNA3.1‐HIP1R group, miR‐92a‐3p mimic group, miR‐92a‐3p mimic + pcDNA3.1‐HIP1R group). (C) Cell proliferation abilities in different groups (NC group, miR‐92a‐3p mimic group, miR‐92a‐3p mimic + Rigosertib (a PI3K/AKT inhibitor)) were investigated by CCK8 assay. (D) The apoptotic events in cells with different treatments (NC group, miR‐92a‐3p mimic group, miR‐92a‐3p mimic + Rigosertib) were quantified by flow cytometry. (E) The relative level of p‐Akt in the xenograft tissues was measured by IHC staining. *p < 0.05, **p < 0.01, ***p < 0.001

To understand the mechanisms by which HIP1R modulates the malignancy of PAAD cells, we performed GSEA (Gene Set Enrichment Analysis) (http://www.gsea‐msigdb.org/gsea/index.jsp), which indicates that high HIP1R expression is negatively associated with PI3K/Akt pathway (Figure 7A). We therefore performed Western blot to examine the relative activation status of PI3K/Akt signalling pathway by measuring the phosphorylation level of Akt, mTOR, S6K and S6 in cells of different groups (NC, HIP1R overexpression, miR‐92a‐3p mimic miR‐92a‐3p mimic + HIP1R overexpression). The results showed that HIP1R overexpression suppressed the phosphorylation of Akt, mTOR, S6K and S6, while miR‐92a‐3p mimic promoted their phosphorylation. The co‐transfection of miR‐92a‐3p mimic suppressed the effect of HIP1R overexpression (Figure 7B). Using Rigosertib (a PI3K/AKT inhibitor), we further demonstrated the effects of miR‐92a‐3p mimic on cell proliferation and apoptosis were largely abrogated by Rigosertib (Figure 7C,D). Besides, miR‐92a‐3p mimic also increased the phosphorylation of AKT in the xenograft tumour samples, while miR‐92a‐3p inhibitor decreased the level of p‐AKT (Figure 7E). Collectively, these results indicate that miR‐92a‐3p/HIP1R axis regulates the malignant progression of PAAD by targeting PI3K/AKT signalling pathway.

## 4 DISCUSSION

PAAD is a common tumour of the digestive system with a high degree of malignancy and a poor prognosis. More than half of PAADs are located in the head of the pancreas, and most of PAADs are ductal adenocarcinomas originating from the epithelium of the pancreatic ducts.^25^, ^26^ It is a big challenge for the early diagnosis of PAAD and the mortality rate remains high after surgical resection or chemotherapies.^27^ Previous studies have suggested that functional restoration of tumour suppressor genes are attractive strategies to curb the progression of cancer.^9^ HIP1R belongs to an evolutionarily conserved family of proteins implicated in the regulation of cell proliferation.^28^, ^29^ It has been reported that HIP1R acts as a tumour suppressor to limit cancer progression in gastric cancer and colorectal cancer.^11^, ^14^ In this study, we also found the upregulation of HIP1R in PAAD tissues and cells, and the overexpression of HIP1R suppressed the cell proliferation, migration and invasion and induced cell apoptosis, while HIP1R silencing impaired the malignant phenotype of PAAD cells. These data suggest that HIP1R also function as a tumour suppressor gene in PAAD.

Epigenetic changes, such as altered patterns of genomic DNA methylation, are associated with human malignancies.^30^ In the past 20 years, the aberrant methylation of CpG islands in the gene promoters was frequently reported in cancers,^31^, ^32^ which predominantly affect the expression of tumour suppressor genes. Because abnormal methylation is one of the earliest molecular changes, it has been considered as a biomarker for early cancer diagnosis.^33^ For example, in clear renal cell carcinoma, hypermethylation of the tumour suppressor gene ADAMTS18 facilitates the proliferation, migration and invasion of tumour cells.^34^, ^35^ A non‐coding RNA HOTAIR could induce the promoter DNA methylation of tumour suppressor gene PCDH10 in gastric cancer and modulate DNMT1 expression by sponging miR‐148b.^36^ In PAAD, the levels of DNA methylation at tumour suppressor genes such as ADAMTS18 and HPP1 genes in cancer tissues are also higher than that in normal tissues.^37^, ^38^ In this study, we found that the CpG islands at the promoter region of HIP1R were hypermethylated in PAAD tissues and cell lines. Importantly, the treatment of a DNA methylation inhibitor 5‐AZA could increase the protein and mRNA expression of HIP1R. Moreover, 5‐AZA treatment impaired the malignancy of PAAD cells in a HIP1R‐dependent manner. Together, these results indicate that the silencing of HIP1R in PAAD tissues is induced by DNA hypermethylation, and demethylation reagent undermines the malignancy of PAAD cells. The DNA hypermethylation is correlated with the upregulation of several DNA methyltransferases, and future works are needed to understand how these methyltransferases are upregulated in PAAD.

Another post‐transcriptional regulation mechanism which is frequently disrupted is the aberrant expression of non‐coding RNAs. Non‐coding RNAs such as miRNAs participate in a wide range of biological processes, including proliferation, migration, metabolism, apoptosis, and epithelial‐mesenchymal transition.^39^ miRNAs usually interact with the target mRNAs and degrade the mRNAs or blocks the translation by binding to the 3' UTR.^39^ The dysregulation of miRNAs has also been reported in PAAD. For instance, Lv et al reported that miR‐4668‐5p is overexpressed in serum samples of PAAD patients,^40^ and miR‐324‐5p upregulation could promote cell proliferation in PAAD cells by suppressing the protein expression of KLF3.^41^ Besides, miR‐887‐3p is also upregulated in PAAD, which promotes the malignant progression of PAAD by down‐regulating STARD13.^42^ In this study, we found that miR‐92a‐3p was upregulated in tumour tissues and cells of PADD, and miR‐92a‐3p overexpression using miR‐92a‐3p mimic enhanced the cell proliferation, migration and invasion and suppressed apoptosis in PAAD cells. These findings seem to be consistent with previous studies in which miR‐92a‐3p were reported as an oncogenic factor to mediate the malignant progression in oesophageal squamous cell cancer, glioma, liposarcoma and hepatocellular carcinoma.^43^, ^44^, ^45^, ^46^ Moreover, this study further demonstrated that miR‐92a‐3p augmented the malignancy of PADD cells by negatively regulating HIP1R and could promote the tumorigenesis of PAAD cells in vivo. We also showed that miR‐92a‐3p/HIP1R axis modulates the activation status of PI3K/Akt signalling. The inhibition of miR‐92a‐3p suppresses the malignancy of PAAD cells by targeting PI3K/Akt pathway. However, the DNA methylation seems not to affect the expression of miR‐92a‐3p, and it remains to be further investigated what are the mechanisms underlying the upregulation of miR‐92a‐3p in PAAD.

In summary, this study demonstrated that HIP1R acts as a tumour suppressor which is downregulated in PAAD tissues and cells, and its reduced expression predicts a poor prognosis in PADD patients. We further elucidate that both DNA hypermethylation at the promoter region of HIP1R and the upregulation of miR‐92a‐3p contributes to the repression of HIP1R expression. These findings add novel insights into the functions of HIP1R in PAAD, which are of valuable clinical significance: HIP1R could serve as a potential prognostic marker in PAAD, and targeting DNA methylation and miR‐92a‐3p could be used as a strategy to restore the tumour suppressor function of HIP1R to curb the progression of PAAD. Future efforts are required to assess whether targeting DNA methylation and miR‐92a‐3p could synergize with the existing chemotherapeutics to improve the treatment outcome.

## AUTHOR CONTRIBUTIONS

**Huiting Xu:** Data curation (equal); formal analysis (equal); methodology (equal); writing – original draft (equal). **Runzhi Chen:** Methodology (equal); software (equal). **Qian Shen:** Methodology (equal); software (equal). **Dongmei Yang:** Methodology (equal); software (equal). **Hui Peng:** Methodology (equal); software (equal). **Jin Tong:** Methodology (equal); software (equal). **Qiang Fu:** Conceptualization (equal); funding acquisition (equal); writing – review and editing (equal).

## FUNDING INFORMATION

This work was supported by the National Natural Science Foundation of China (No. 81974381).

## CONFLICT OF INTERESTS

The authors declare no competing financial interest.

## PATIENT CONSENT FOR PUBLICATION

Informed consent was obtained from all individual participants included in the study.

## Supporting information


Figure S1

Figure S2

Figure S3

Figure S4

Appendix S1

## Data Availability

The datasets used and/or analyzed during the current study are available from the corresponding author on reasonable request.
