# Supplementary figures and images for: DNA methylation and miR‐92a‐3p‐mediated repression of HIP1R promotes pancreatic cancer progression by activating the PI3K/AKT pathway
Source: J Cell Mol Med. 2023 Feb 21;27(6):788–802. doi: 10.1111/jcmm.17612 (PMC10002968; doi:10.1111/jcmm.17612)

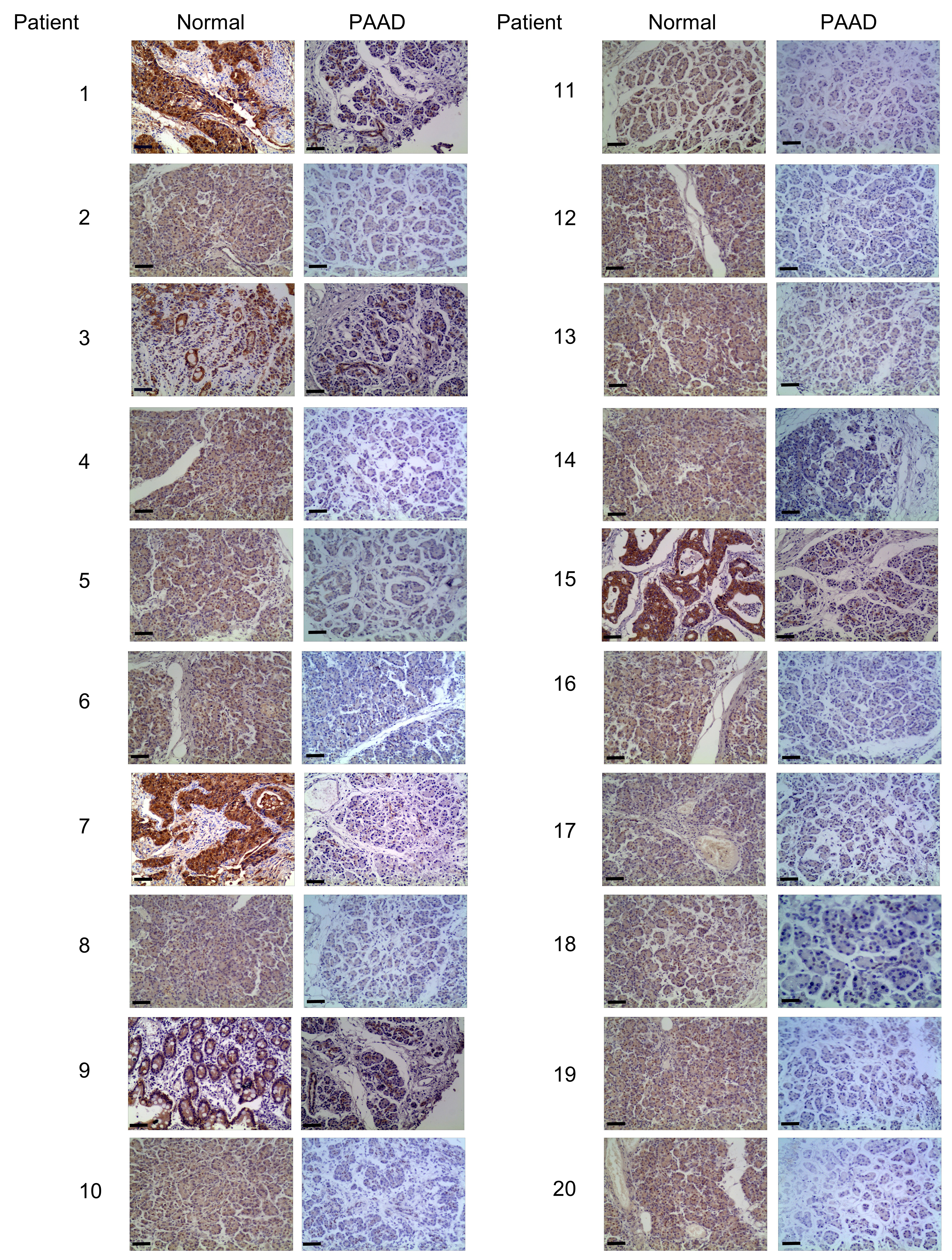

Supplement: Supplementary file 1 — Figure S1 [file JCMM-27-788-s002.jpg]

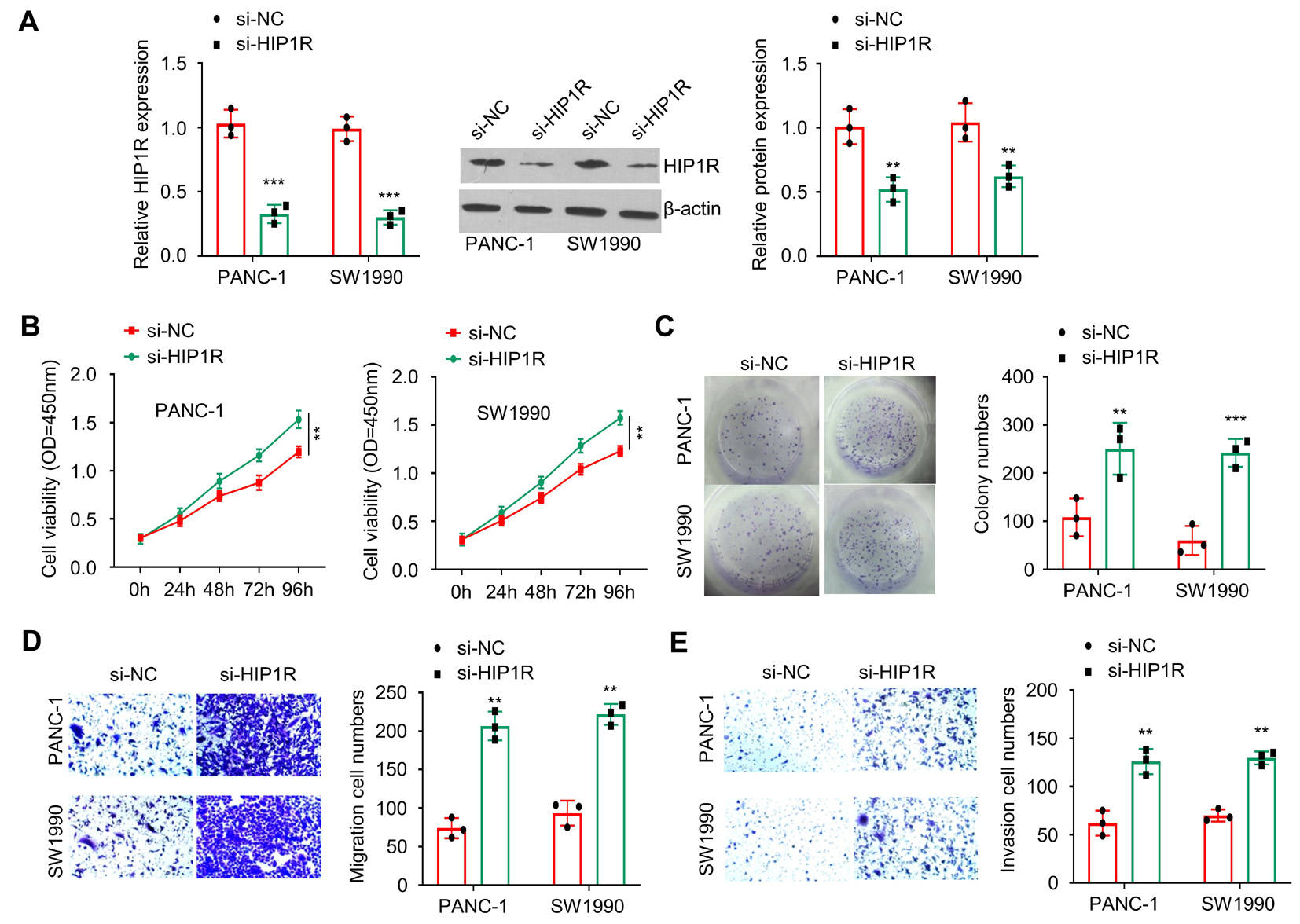

Supplement: Supplementary file 2 — Figure S2 [file JCMM-27-788-s005.jpg]

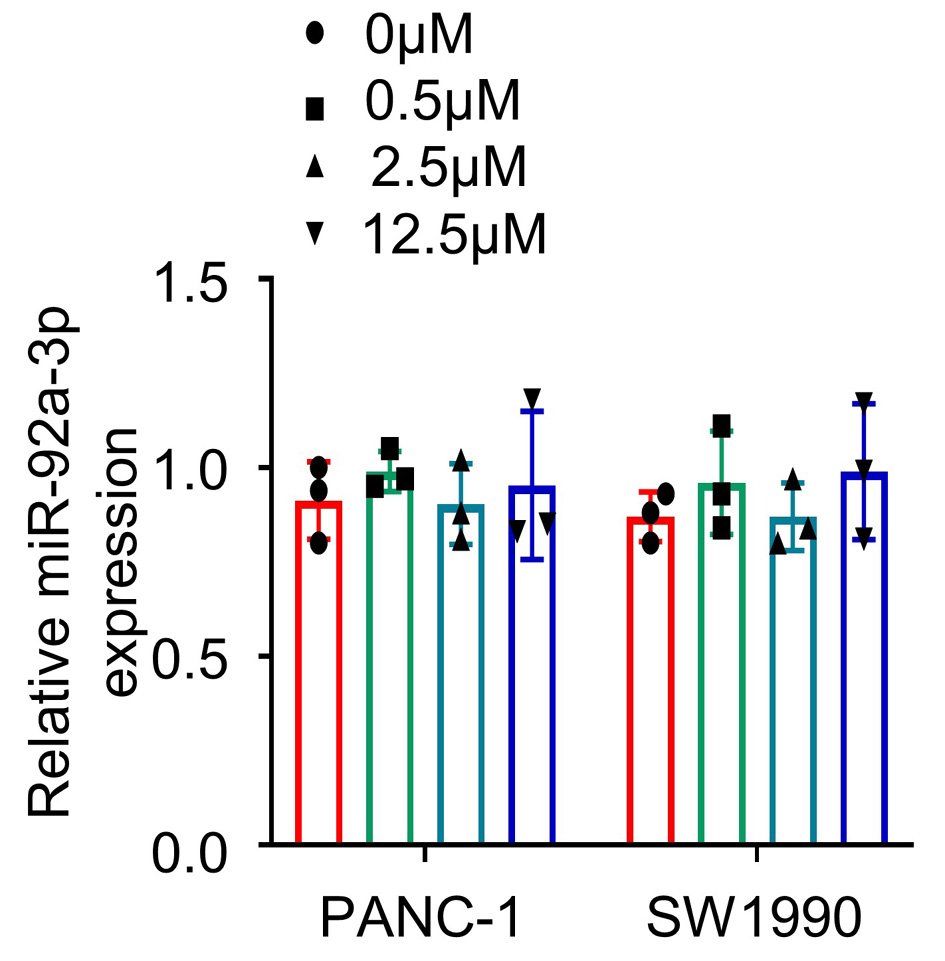

Supplement: Supplementary file 3 — Figure S3 [file JCMM-27-788-s001.jpg]

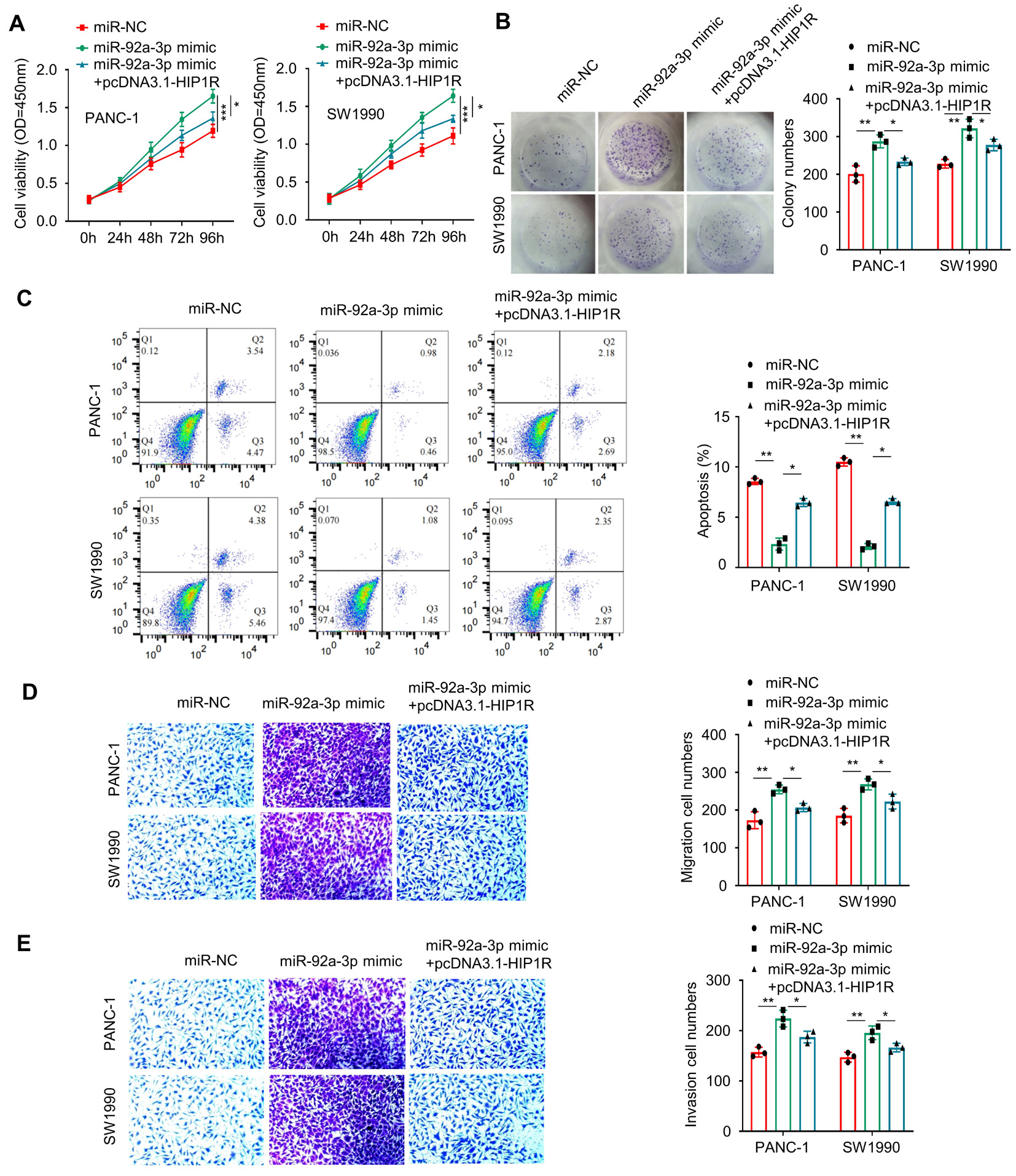

Supplement: Supplementary file 4 — Figure S4 [file JCMM-27-788-s004.jpg]
