# Supplementary material for: DNA methylation and miR‐92a‐3p‐mediated repression of HIP1R promotes pancreatic cancer progression by activating the PI3K/AKT pathway
Source: J Cell Mol Med. 2023 Feb 21;27(6):788–802. doi: 10.1111/jcmm.17612 (PMC10002968; doi:10.1111/jcmm.17612)
Supplement: Supplementary file 5 — Appendix S1 [file JCMM-27-788-s003.docx]

Supporting Information: Figure Legends

Supplementary Figure 1 (A-B) The protein level of HIP1R PAAD tumour tissues and the matched para-cancerous normal tissues were analysed by IHC staining. Images showed the representative IHC staining of HIP1R tissues from 20 patients (including the samples shown in Figure 1) (scale bar: 50 μm).

Supplementary Figure 2 (A) The mRNA and protein levels of HIP1R in PANC-1 and SW1990 cells were examined after the transfection of control si-RNA (si-NC) or siRNA targeting HIP1R (si-HIP1R). (B) Cell proliferation of PAAD cells in different groups (si-NC and si-HIP1R) was assessed by CCK8 assay. (C) Clonogenic abilities of PAAD cells in different groups (si-NC and si-HIP1R) were determined by colony formation assay. (D-E) Cell migration and invasion abilities of PAAD cells in different groups (si-NC and si-HIP1R) were examined by Transwell migration and invasion assays. **p* < 0.05, ***p* < 0.01, ****p* < 0.001.

Supplementary Figure 3 The relative expression levels of miR-92a-3p were examined by qRT-PCR in PANC-1 and SW1990 cells after the treatment of different doses of 5-AZA.

Supplementary Figure 4 (A) Cell proliferation abilities of PAAD cells with different treatments (miR-NC group, miR-92a-3p mimic group, miR-92a-3p mimic+ pcDNA3.1-HIP1R group) were examined by CCK8 assay (B) The clonogenic abilities of cells in different groups (miR-NC group, miR-92a-3p mimic group, miR-92a-3p mimic+pcDNA3.1-HIP1R group) were determined by colony formation assay. (C) The apoptotic events of PAAD cells with different treatments (miR-NC group, miR-92a-3p mimic group, miR-92a-3p mimic+pcDNA3.1-HIP1R group) were quantified by flow cytometry. (D-E) Cell migration and invasion abilities in different groups of cells (miR-NC group, miR-92a-3p mimic group, miR-92a-3p mimic+pcDNA3.1-HIP1R group) were assessed by Transwell assay. **p* < 0.05, ***p* < 0.01, ****p* < 0.001
